# Enhanced central sympathetic tone induces heart failure with preserved ejection fraction (HFpEF) in rats

**DOI:** 10.3389/fphys.2023.1277065

**Published:** 2023-12-07

**Authors:** Shyam S. Nandi, Kenichi Katsurada, Michael J. Moulton, Hong Zheng, Kaushik P. Patel

**Affiliations:** ^1^ Department of Cellular and Integrative Physiology, University of Nebraska Medical Center, Omaha, NE, United States; ^2^ Division of Cardiovascular Medicine, Department of Internal Medicine, Jichi Medical University School of Medicine, Shimotsuke, Tochigi, Japan; ^3^ Department of Surgery, University of Nebraska Medical Center, Omaha, NE, United States; ^4^ Basic Biomedical Sciences, Sanford School of Medicine, University of South Dakota, Vermillion, SD, United States

**Keywords:** sympatho-excitation, diastolic dysfunction, cardiac remodeling, heart failure, angiotensin II

## Abstract

Heart failure with preserved ejection fraction (HFpEF) is a heterogenous clinical syndrome characterized by diastolic dysfunction, concentric cardiac left ventricular (LV) hypertrophy, and myocardial fibrosis with preserved systolic function. However, the underlying mechanisms of HFpEF are not clear. We hypothesize that an enhanced central sympathetic drive is sufficient to induce LV dysfunction and HFpEF in rats. Male Sprague–Dawley rats were subjected to central infusion of either saline controls (saline) or angiotensin II (Ang II, 20 ng/min, i.c.v) *via* osmotic mini-pumps for 14 days to elicit enhanced sympathetic drive. Echocardiography and invasive cardiac catheterization were used to measure systolic and diastolic functions. Mean arterial pressure, heart rate, left ventricular end-diastolic pressure (LVEDP), and ± dP/dt changes in responses to isoproterenol (0.5 μg/kg, iv) were measured. Central infusion of Ang II resulted in increased sympatho-excitation with a consequent increase in blood pressure. Although the ejection fraction was comparable between the groups, there was a decrease in the E/A ratio (saline: 1.5 ± 0.2 vs Ang II: 1.2 ± 0.1). LVEDP was significantly increased in the Ang II-treated group (saline: 1.8 ± 0.2 vs Ang II: 4.6 ± 0.5). The increase in +dP/dt to isoproterenol was not significantly different between the groups, but the response in -dP/dt was significantly lower in Ang II-infused rats (saline: 11,765 ± 708 mmHg/s vs Ang II: 8,581 ± 661). Ang II-infused rats demonstrated an increased heart to body weight ratio, cardiomyocyte hypertrophy, and fibrosis. There were elevated levels of atrial natriuretic peptide and interleukin-6 in the Ang II-infused group. In conclusion, central infusion of Ang II in rats induces sympatho-excitation with concurrent diastolic dysfunction, pathological cardiac concentric hypertrophy, and cardiac fibrosis. This novel model of centrally mediated sympatho-excitation demonstrates characteristic diastolic dysfunction in rats, representing a potentially useful preclinical murine model of HFpEF to investigate various altered underlying mechanisms during HFpEF in future studies.

## Introduction

Heart failure with preserved systolic function (HFpEF) is a complex, heterogenous cardio-metabolic syndrome where multi-organ comorbidities create hemodynamic alterations such as hypertension with a systemic inflammatory state (Paulus and Tschope, 2013; Pitt et al., 2014). Systemic inflammation leads to downstream effects on the heart and blood vessels and that leads to hypertension, which in turn leads to diastolic dysfunction, concentric or eccentric cardiac hypertrophy, myocardial extracellular matrix expansion/fibrosis, exercise intolerance, and pulmonary congestion, with a preserved systolic function (Upadhya et al., 2015; Gori et al., 2016; Hanff et al., 2021; Yoon et al., 2021). Common comorbidities in HFpEF patients include hypertension, obesity, type 2 diabetes, and renal dysfunction (Altara et al., 2017; Olver et al., 2019; Schiattarella et al., 2019; Nguyen et al., 2020). Of note, it is challenging to model a preclinical animal model that mimics all these basic clinical features of HFpEF. Recent developments of several “multiple hits” models of HFpEF incorporate these comorbidity factors to mimic the clinical condition (Schiattarella et al., 2019; Deng et al., 2021; Sharp et al., 2021). However, the advantage of developing a model independent of these comorbidities but having enhanced sympatho-excitation allows one to also elucidate the possible contributions of other factors, such as renal dysfunction and baroreceptor dysfunction, in the development of HFpEF.

We have chosen to identify one common characteristic which is prevalent among the majority of these comorbidities that may be an all-encompassing abnormality and may be the critical feature for the development of HFpEF; the increased sympathetic nervous activation is the factor which is being observed in chronic heart failure with reduced systolic function (HFrEF), hypertension, obesity, type 2 diabetes, aging, renal dysfunctions, and systemic inflammation (Kaye et al., 1994; Kaye and Esler, 2005; Kalil and Haynes, 2012; Patel et al., 2016; Zheng et al., 2016; Fonkoue et al., 2019; Sharma et al., 2021). Considering the treatment of HFrEF, several clinical trial studies suggest that reduction of sympathetic hyperactivity using pharmacological beta blockers is beneficial (Camara and Osborn, 1999; Barki-Harrington et al., 2004; Little, 2008). We posit that increased sympathetic hyperactivity, perhaps not as massive as that observed in the HFrEF condition, may be the critical factor for the development of HFpEF, gradually over time. Impaired myocardial sympathetic innervation has been reported to be associated with diastolic dysfunction in HFpEF (Aikawa et al., 2017).

We have recently demonstrated that central administration of angiotensin II (Ang II) elicits a sympatho-excitatory state, resulting in hypertension and activation of the sympathetic tone to the heart (Sharma et al., 2021). Recent literature suggests that central regulation of sympathetic outflow may be mediated by the brain renin–angiotensin system (RAS), an imbalanced redox stress axis, and pro-inflammatory cytokines/chemokine dysregulations (Dikalov and Nazarewicz, 2013; Loredo-Mendoza et al., 2020; Sava et al., 2020). These central RAS mechanisms have also been implicated in the development and progression of heart failure in general. The present study proposes to examine the hypothesis that specific activation of central sympatho-excitatory pathways by the administration of Ang II centrally elicits a sympatho-excitatory state, resulting in hypertension, and mimics several phenotypic abnormalities, which is unique to the pre-clinical experimental rat model of HFpEF.

## Materials and methods

### Animals and treatments

Sprague–Dawley rats (male rats: 250–300 g, ∼12 weeks old) from the SASCO Laboratory were housed in the central BSL3 animal facility. Rats were housed in a hygienic BSL3 room maintained at 30%–40% humidity, 22°C–24°C air temperature, with 12 h of diurnal cycles, and on normal rat chow and water provided *ad libitum*. All experimental protocols, methods, and handling of animals in this study were approved by the University of Nebraska Medical Center Institutional Animal Care and Use Committee (IACUC).

Rats were randomly assigned to either of two groups: intracerebroventricular (ICV) infusion of isotonic saline (saline group) or the Ang II group with ICV infusion of Ang II *via* osmotic mini-pumps for 14 days to induce continuous sympatho-excitation and hypertension, as shown previously (Supplementary Figures S1A–D, Sharma et al., 2021; Becker et al., 2017; Sharma et al., 2021). For ICV cannulation and mini-pump placement surgery, rats were anesthetized with a mixture of ketamine (87 mg/kg) and xylazine (10 mg/kg), i.p. injection. The bregma was exposed with a small skin incision on the head, and a small hole was made in the skull bone to access the dura. ICV cannula (ALZET Brain Infusion Kit 1) was secured and cemented on the skull, and directed to the third ventricle using the stereotaxic coordinates of Paxinos and Watson atlas (Paxinos et al., 1980) (1.5 mm lateral to the midline, 4.0 mm ventral to the cranium, and 0.8 mm caudal to the bregma), as described previously (Sharma et al., 2021). The brain cannula was connected for ICV infusion from the osmotic minipump (ALZET, model 2002) for Ang II (20 ng/min) or sterile isotonic saline as vehicle control for 14 continuous days, as described previously (Becker et al., 2017; Sharma et al., 2021). Rat groups were blinded for physiological recording and echocardiographic evaluations as well as invasive cardiac pressure measurements at the end of the infusion period. Ang II-infused rats that demonstrated a dipsogenic response were included in the study. Ang II-infused rats which did not demonstrate a dipsogenic response were considered not to have viable Ang II infusion and, therefore, were excluded from the study (n = 1). Inclusion and exclusion criteria were set before the study. At the end of the experiment, the brains were serially sectioned and the placement of the cannula in the brain was identified. One animal excluded with no dipsogenic response was identified to have the brain cannula in the parenchyma of brain tissue and not in the third ventricular space. Statistical methods are described in the main text.

### M-mode echocardiography and Doppler imaging

Before the start of the experiment on day −2 and at the end of 12 days of ICV treatment, cardiac hemodynamic parameters were accessed using M-mode echocardiography (using Vevo 3100 Imaging System, VisualSonics) with a transducer (Supplementary Figure S1). In brief, anesthesia was induced by a 2% isoflurane chamber and confirmed by no hind limb muscle reflex response. During echocardiography acquisition, isoflurane was maintained at 2%. B-mode echo images were captured in the parasternal long-axis plane, and M-mode images were captured at the level of the papillary muscles of the left ventricle (LV) represented as the mid-ventricular position. The LV end-systolic diameter (LVESd), LV end-diastolic diameter (LVEDd), LV ejection fraction (LVEF%), fractional shortening (FS), and LV volumes were measured and calculated using standard formulas from the Vevo LAB software, VisualSonics. The peak Doppler blood flow velocities across the mitral valve during early and late diastole were also captured. LV mitral filing parameters (E/A) were measured and compared with the respective baseline for the assessment of diastolic dysfunction in both groups of rats. The person performing the echocardiography acquisition and analyses was blinded to all animal groups.

### Direct *in vivo* hemodynamic measurements

At the end of 14 days of ICV treatments, hemodynamic parameters were accessed using a Mikro-Tip catheter (SPR-407, Millar Instruments; Houston, TX) introduced into the LV in an anesthetized non-survival terminal procedure in an experimenter-blinded fashion. In brief, animals were anesthetized using a single injection of urethane (0.75 g/kg i.p.) and chloralose (60 mg/kg i.p.). The catheter was inserted into the LV chamber *via* the right carotid artery, as described previously (Nandi et al., 2016). The hemodynamic parameters, mean arterial pressure (MAP), and heart rate (HR) were simultaneously recorded on the PowerLab Data Acquisition System (8SP, ADInstruments) and analyzed as reported previously (Nandi et al., 2016; Patel et al., 2016). The cardiac contractile responsiveness (change in ±dP/dt) to adrenergic agonist was evaluated by intravenous infusion of two doses of isoproterenol (β-adrenoreceptor agonist, 0.1 and 0.5 μg/kg). At the end of the experiment, rats were euthanized using Fatal Plus euthanasia solution (120 mg/kg pentobarbital, i.p.).

### Wheat germ agglutinin staining

Wheat germ agglutinin (WGA) staining of 5 µm transverse sections was performed on cryosections of the heart using a CryoStar NX50 (Thermo Fisher Scientific). Sections were fixed in 4% paraformaldehyde solution for 20–30 min. Sections were washed twice in TBS, 5 min each after post-fixation, and incubated in the dark with WGA staining solution (5 mg/mL, Thermo Fisher Scientific) for 15 min at room temperature. Following incubation of the sections, they were washed thrice in TBS and mounted with a coverslip. WGA fluorescence images were captured using a fluorescence microscope (Olympus IX71 Imaging Systems). The cardiomyocyte diameter was determined by measuring 50 cells per LV section, and three LV sections were evaluated for each heart. A total of 150 cells were measured per heart. The cardiomyocyte diameter and numbers per unit area from the WGA-stained images were evaluated in an observer-blinded fashion.

### Masson’s trichrome staining

To determine cardiac perivascular and interstitial fibrosis, we performed Masson’s trichrome staining, where blue staining denotes collagen fibers. Masson’s Trichrome Kit (Thermo Fisher Scientific) was used to stain 5 µm paraffin longitudinal sections of the heart. Perivascular fibrosis and interstitial fibrosis were determined by evaluating 20 fields per LV section, and three LV sections were evaluated for each heart. We calculated perivascular and interstitial fibrosis by quantifying the % blue pixel intensity normalized to the total area pixel intensity using the color deconvolution tool of Fiji ImageJ software, NIH. The Tissue Core Facility service of the University of Nebraska Medical Center was used for the Masson’s trichrome staining procedure. Quantification of the slides was performed in an experimenter-blinded fashion.

### Picrosirius red staining

Masson’s trichrome staining results were corroborated with those of Picrosirius red staining. In brief, 10% formalin-fixed LV paraffin sections (5 µm) were processed for Picrosirius red staining. The reagents were Direct Red 80, picric acid, and glacial acetic acid. The Tissue Core Facility service of the University of Nebraska Medical Center was used for the Picrosirius red staining procedure. Quantifications were performed in an experimenter-blinded fashion.

### Western blot analysis

Western blot analyses were performed to measure the level of proteins from heart tissues following our previously published protocols (Nandi et al., 2020; Nandi et al., 2021). In brief, heart tissues were homogenized in RIPA buffer and whole tissue protein extracts were prepared by centrifugation at 4°C. The protein concentration was measured by the BCA method of protein assay (Thermo Fisher Scientific). Denatured protein samples were loaded on SDS-PAGE, and resolved protein gels were transferred onto a PVDF (polyvinylidene fluoride) membrane. The transferred PVDF membranes were blocked in 5% milk (non-fat dried milk, Bio-Rad) in TBS for 60 min at room temperature and washed thrice in TBS wash buffer for 15 min. Membranes were then incubated overnight at 4°C with 1:1000 diluted primary antibodies using TBS as a dilution buffer. The primary antibodies used were atrial natriuretic peptide (ANP, cat# GTX109255, GeneTex) and interleukin-6 (IL-6, cat# AB9324, Abcam). HRP-mouse or HRP-rabbit secondary antibodies were diluted at 1:4000 in TBS as a diluent buffer and incubated for 2 h at ambient temperature. A SuperSignal^®^ West Femto stable peroxidase buffer chemiluminescent kit (cat# 1859023, Thermo Scientific) was used for Western blot band detections using the ChemiDoc™ XRS Molecular Imager (Bio-Rad Laboratories). The images were captured using the Image Lab software version 6, and quantified and normalized from respective loading controls (Bio-Rad Laboratories).

### Statistical analysis

Data are expressed as mean ± SE. Differences between the groups were assessed by *t*-test analysis of significance (Prism 7; GraphPad Software) as appropriate. A *p*-value <0.05 was considered indicative of statistical significance.

## Results

### General morphological and hemodynamic characteristics in ICV Ang II-infused rats

The general morphological and hemodynamic characteristics at baseline of the two groups of rats used in this study are summarized in Table 1. As expected, Ang II infusion resulted in increased mean arterial pressure in anesthetized conditions, which is consistent with our previous report of an increase in arterial blood pressure and enhanced cardiac and renal sympathetic tone (Sharma et al., 2021). The heart weight and heart weight/body weight ratio were significantly higher in the Ang II-infused group than in the saline-infused group. The Ang II group also demonstrated elevated lung weight indicative of pulmonary congestion.

**TABLE 1:** General characteristics of rats treated with saline (i.c.v) or Ang II (i.c.v).

Parameters	Saline (i.c.v) (n = 6)	Ang II (i.c.v) (n = 6)
*Gravimetric measurements*		
BW (g)	341 ± 11	260 ± 07*
HW (mg)	936 ± 10	1117 ± 49*
HW/BW (g/mg)	2.86 ± 0.03	3.98 ± 0.18*
TL (cm)	4.90 ± 0.19	4.91 ± 0.22
HW/TL (mg/cm)	186 ± 4	248 ± 12*
LW(g)	1.53 ± 0.04	1.75 ± 0.05*
LW(g)/TL (cm)	0.34 ± 0.01	0.40 ± 0.01*
*Invasive hemodynamic measurements* (*baseline values under anesthesia*)		
HR (bpm)	375 ± 11	380 ± 63
MAP	83 ± 3	123 ± 8*
+dP/dt (mmHg/s)	8,236 ± 801	7,631 ± 897
-dP/dt (mmHg/s)	7,689 ± 501	7,179 ± 730
LVEDP (mmHg)	1.8 ± 0.2	4.6 ± 0.5*
*Echocardiographic measurements*		
HR (bpm)	348 ± 19	346 ± 14
LVESd (mm)	3.85 ± 0.51	4.13 ± 0.48
LVEDd (mm)	7.33 ± 0.54	7.63 ± 0.57
LVPW,d (mm)	1.64 ± 0.29	1.97 ± 0.21
LV mass (mg)	742.7 ± 81.7	1008.2 ± 81.9*
%LVEF	77.4 ± 4.2	75.7 ± 3.1
%LVFS	48.7 ± 4.7	46.4 ± 2.7
E/A	1.47 ± 0.10	1.21 ± 0.06*
E/e’	14.32 ± 0.58	12.91 ± 0.62

BW, body weight; HW, heart weight; TL, tibia length; LVEF, left ventricular ejection fraction; LVFS, left ventricular fractional shortening; LW, lung weight; LVEDP, left ventricular end-diastolic pressure; MAP, mean arterial pressure; HR, heart rate; LVID, left ventricular internal dimension; LVPW, left ventricular posterior wall; E/A, transmitral flow peak velocities; E/e', transmitral tissue Doppler flow peak velocities; i.c.v, intracerebroventricular. Data are represented as mean ± SE of six rats in each group. *p < 0.05 sham vs HFpEF. *p < 0.05 sham vs. HFpEF, Student’s t-test.

### Diastolic dysfunction assessed using echocardiography

**FIGURE 1:**
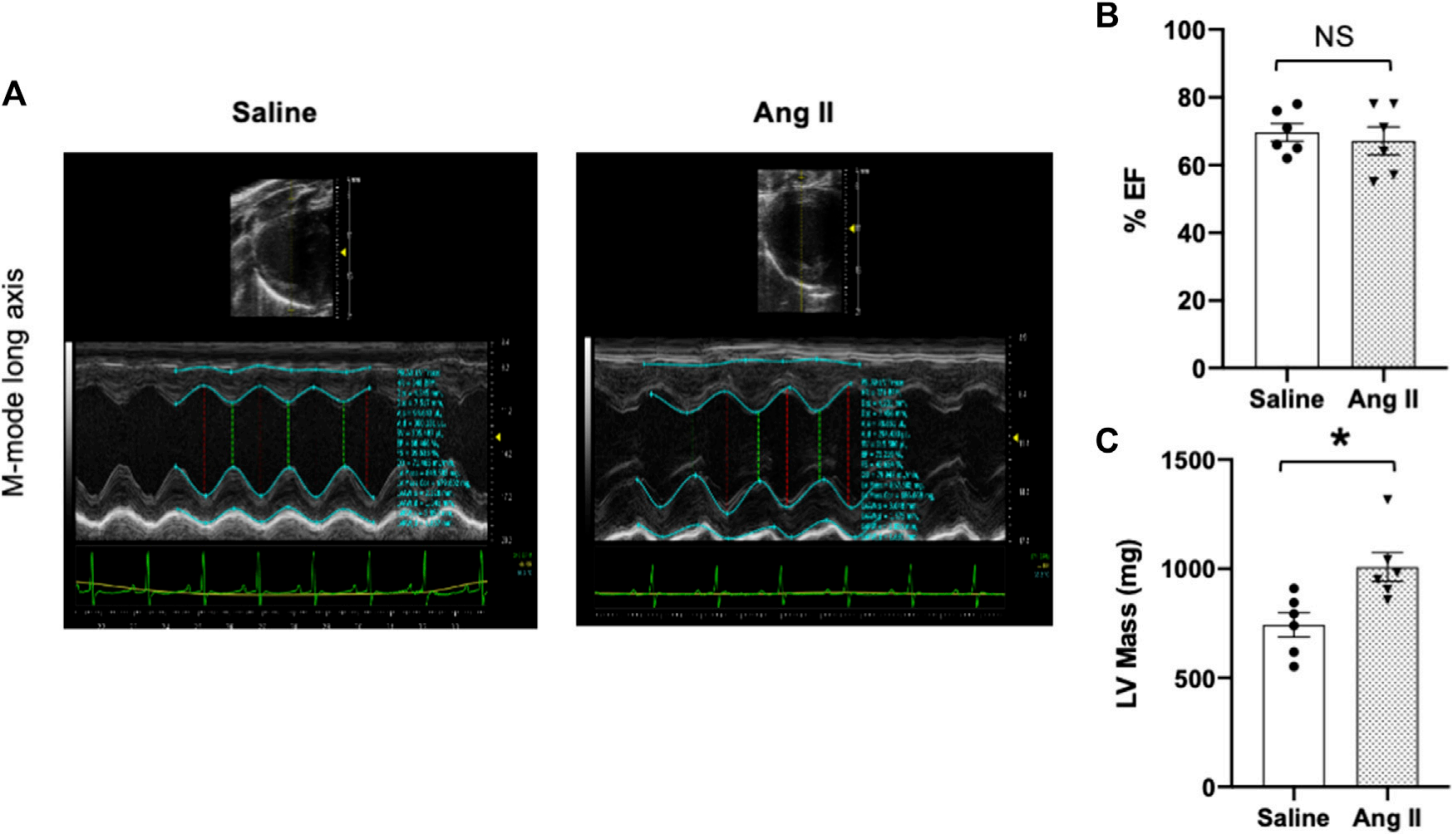
Ang II heart showed increased left ventricular wall remodeling with preserved ejection fraction. **(A)** Representative left ventricular (LV) M-mode echocardiographic tracings. **(B)** Measurements of LV percentage ejection fraction (%EF). **(C)** Measurement of LV mass. Values are presented as mean ± SEM, n = 6. *p < 0.05 vs saline, Student’s t-test.

**FIGURE 2:**
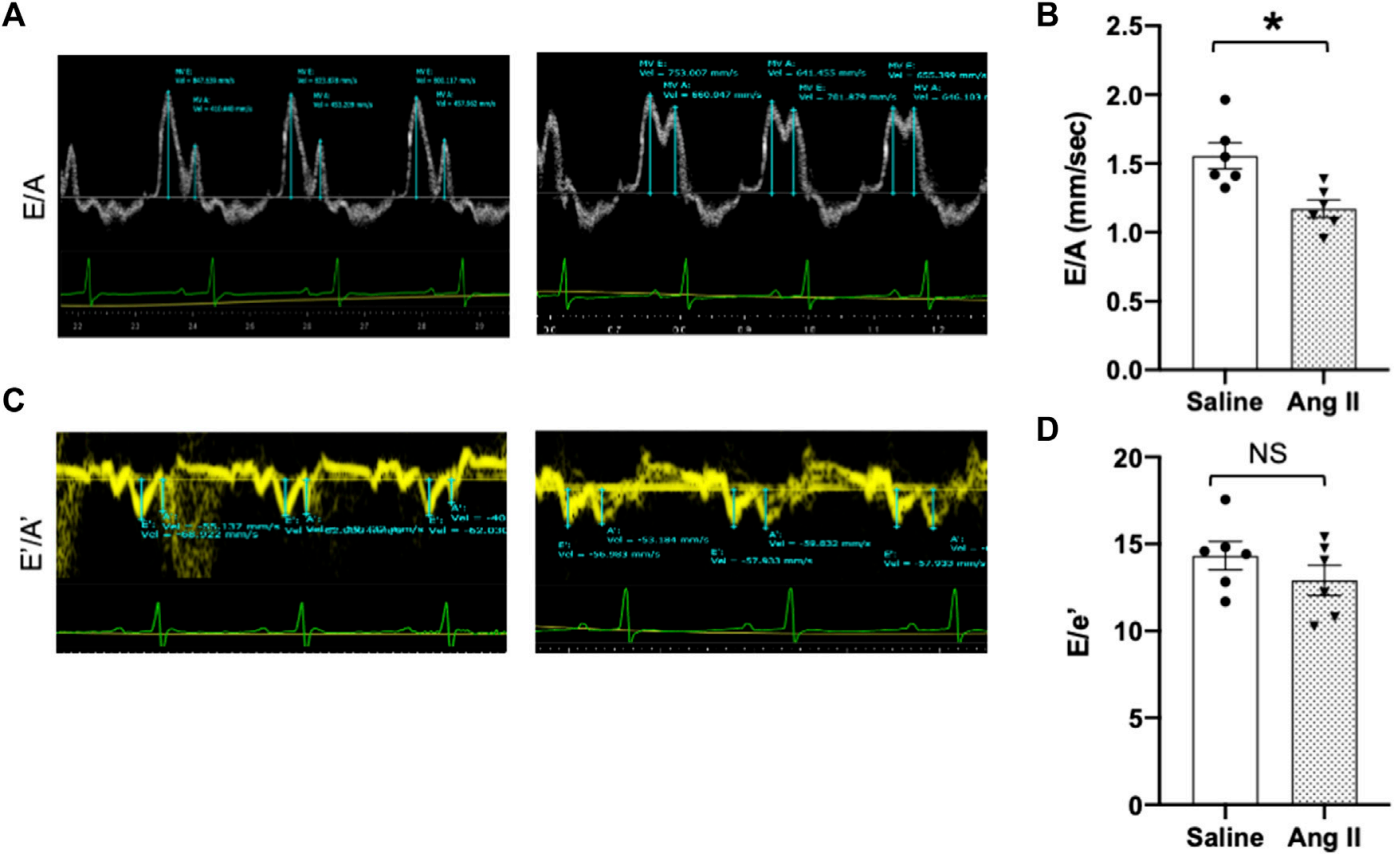
Ang II heart showed reduced early-to-late diastolic transmitral flow velocity (E/A) ratio. **(A)** Representative pulsed-wave Doppler imaging showed E and A tracings. **(B)** Measurement of E/A. **(C)** Tissue Doppler image tracings showing E′ and A’. **(D)** Measurement of E/e’. Values are presented as mean ± SEM, n = 6. *p < 0.05 vs saline, Student’s t-test.

To monitor the changes in cardiac systolic and diastolic functions, we performed noninvasive echocardiographic evaluation with Doppler imaging before (baseline) and after ICV infusion of either saline or Ang II for 14 days (Figure 1; Figure 2) in lightly anesthetized rats. The long-axis echocardiographic evaluation revealed a persistent preservation of the percentage LV ejection fraction (%EF) in both groups of rats (Figure 1B). Rats continuously exposed to ICV Ang II for 14 days manifested increased LV mass (Figure 1C). Furthermore, mitral inflow patterns demonstrated diastolic dysfunction in the Ang II-infused group (E/A, saline: 1.47 ± 0.10 vs Ang II: 1.21 ± 0.06) (Figure 2). This was due to a combination of a reduced E and a slight increase in A, perhaps related to enhanced flow from the atria to fill the ventricle. As we had echography measurements before and after central infusions in each group, we also performed the paired comparisons within each group, and the results showed that there was a significant reduction in the Ang II group but not in the saline group. The heart rates were similar in the two groups. Overall, our echocardiography analyses showed that there was significant LV diastolic dysfunction in the Ang II-infused group, as evidenced by reductions in the E/A ratio of mitral filings; however, LV ejection fraction was preserved (Table 1).

### Diastolic dysfunction assessed using left ventricular catheterization

**FIGURE 3:**
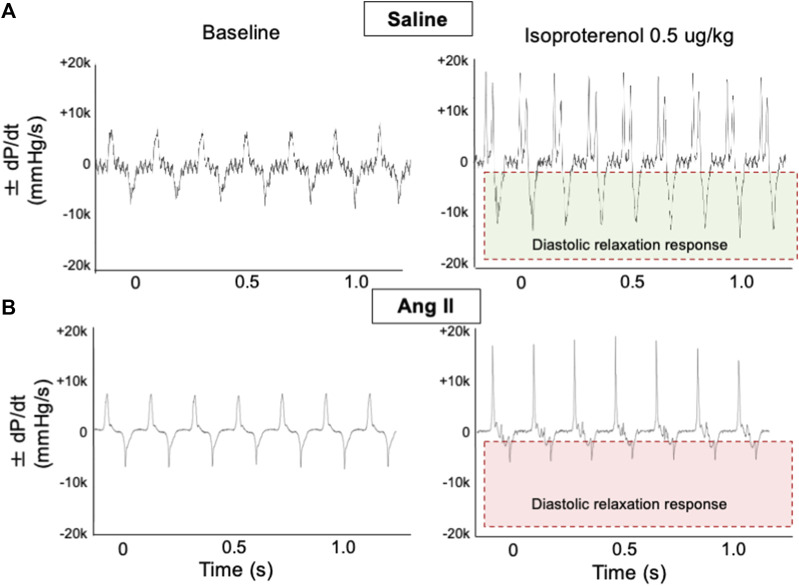
Ang II heart showed reduced diastolic relaxation. Representative dP/dt recordings in hearts from ICV saline- **(A)** and Ang II-treated **(B)** rats before (baseline) and after administration of adrenergic agonist isoproterenol (0.5 μg/kg iv). Isoproterenol-induced diastolic relaxation response is highlighted with boxes.

**FIGURE 4:**
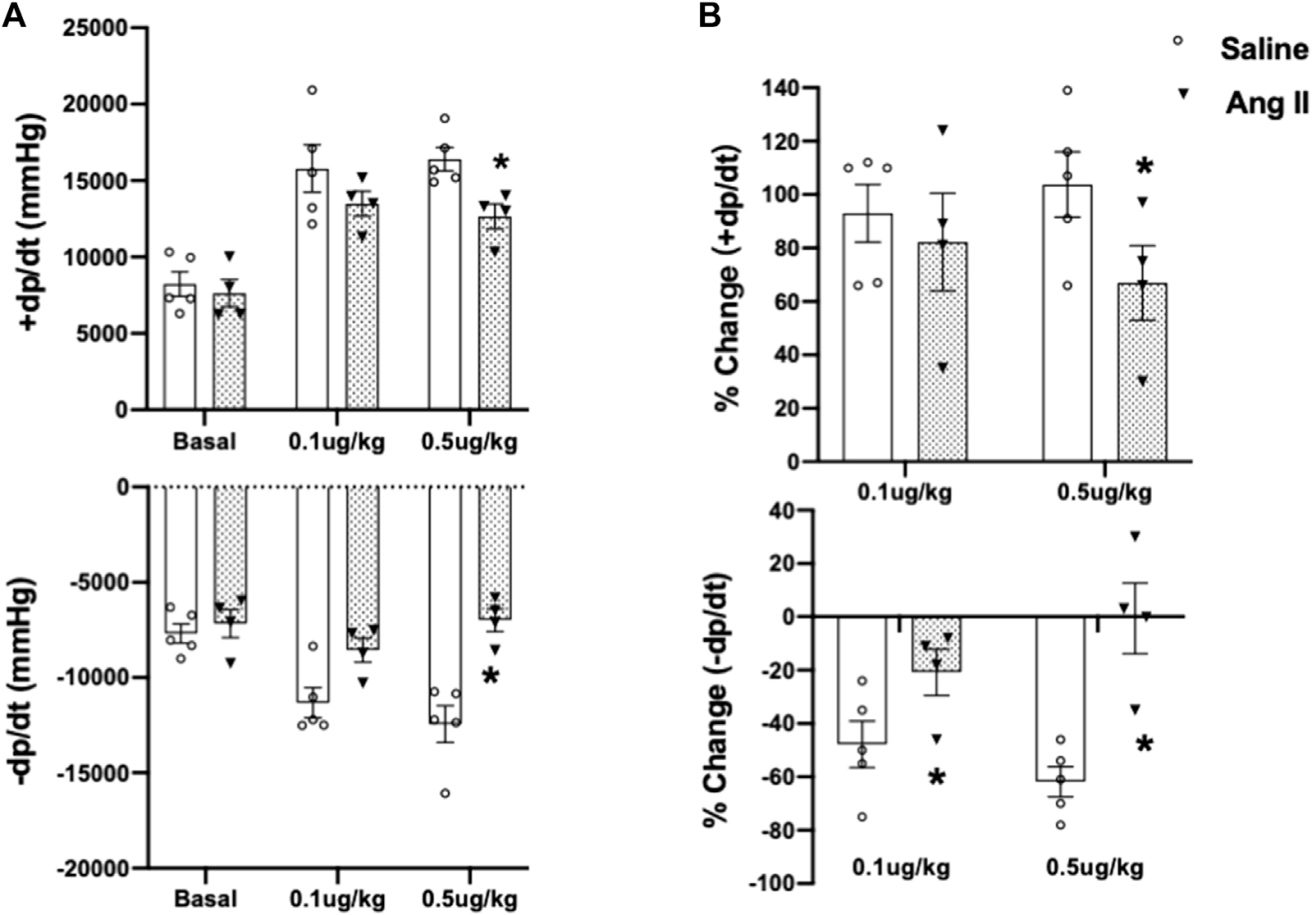
Cumulative quantifications of cardiac hemodynamic recordings using a Millar catheter. **(A)** Mean values for ± dP/dt under basal conditions and during two doses of isoproterenol (0.1 and 0.5 μg/kg iv) in ICV saline- and Ang II-treated rats. **(B)** Percentage change of ±dP/dt to baseline after two doses of isoproterenol (0.1 and 0.5 μg/kg iv) in ICV saline and Ang II-treated rats. Values are presented as mean ± SEM, n = 4–5. *p < 0.05 vs saline.

To further validate cardiac diastolic dysfunction, we catheterized the LV and monitored LV pressure, which is considered the gold standard for monitoring HFpEF. On day 14, terminal hemodynamics measurements using a Millar catheter placed in the LV showed early signs of increased LV filling pressure in the Ang II group (saline: 1.83 ± 0.21 vs Ang II: 4.61 ± 0.47) (Table 1). Furthermore, we examined relaxation indices by measuring the magnitude of active relaxation, change in cardiac negative (diastolic preserve) at the baseline with no perturbation and in the presence of a challenge with isoproterenol, and a β-adrenoreceptor agonist, at two doses (0.1 and 0.5 ug/kg) to induce acute cardiac stress in a subset of the rats (Figure 3; Figure 4). Hemodynamic data monitoring cardiac ± dP/dt demonstrated that cardiac systolic contraction (positive dP/dt) was similar to a normal heart at rest in response to acute stress with isoproterenol, suggesting slight systolic dysfunction in response to acute cardiac stress (Figures 3A, B). However, there is a striking lack of responsiveness in the magnitude of negative dP/dt (-dP/dt), demonstrating a significant reduction in normal cardiac relaxation in response to isoproterenol as an acute cardiac stress inducer (Figures 4A, B).

### Cardiac hypertrophy in ICV Ang II-infused rats

**FIGURE 5:**
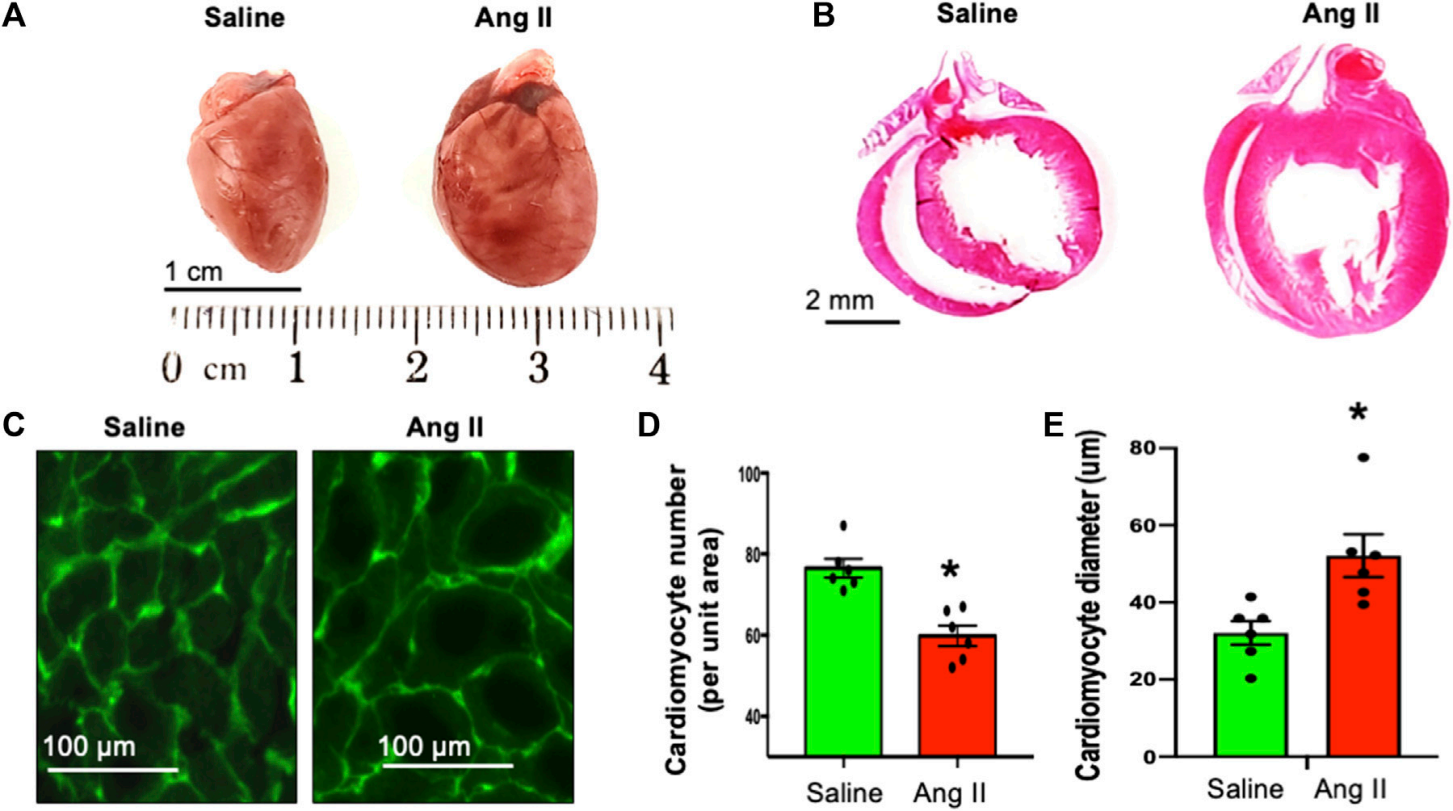
Ang II-infused heart showed cardiac hypertrophy. **(A)** Whole heart morphology of saline- and Ang II-treated rat hearts. **(B)**. H&E stains of whole heart longitudinal sections from saline- and Ang II-treated rats. **(C)** WGA-488 fluorescence images of heart sections from saline- and Ang-treated rat hearts; green fluorescence represents cardiomyocyte boundaries stained with WGA. **(D)** Quantifications of WGA-stained cardiomyocyte numbers per unit area. Each dot represents the average number of cardiomyocytes counted per unit area image of the LV section. **(E)** Quantifications of WGA-stained cardiomyocyte diameter. Each dot represents an average of 150 cardiomyocyte cross-sectional diameters per heart. Values are presented as mean ± SEM, n = 6. *p < 0.05 vs saline, Student’s t-test.

Consistent with the observed elevation in arterial pressures in Ang II-infused rats, they also exhibited an increase in the heart to body weight ratio (Figure 5), indicating hypertrophic cardiac remodeling possibly due to the increased arterial pressure. To characterize the morphological features of cardiac hypertrophic remodeling, we measured the cardiac morphometry of the heart. Morphometric comparison of whole hearts revealed a gross increase in the overall cardiac size in rats in the Ang II group (Figure 5A). To get corroborating cardiac hypertrophy data at the histological level, we performed H&E staining of whole heart sections to visualize the histology of atrial and ventricular chambers. Our results corroborated an increase in the gross heart size and thickening of the LV wall in rats infused with Ang II (Figure 5B). We further evaluated cardiac hypertrophy at the level of cellular cardiomyocytes. Although we observed a reduction in the cardiomyocyte number per unit area, there was an increase in cardiomyocyte diameter per unit area in rats infused with Ang II. This is a further indication of cellular hypertrophy in the hearts of rats infused with Ang II (Figures 5C–E). Overall, these data indicate cardiac hypertrophy with remodeling in rats with Ang II infusion.

### Cardiac fibrosis in ICV Ang II-infused rats

**FIGURE 6:**
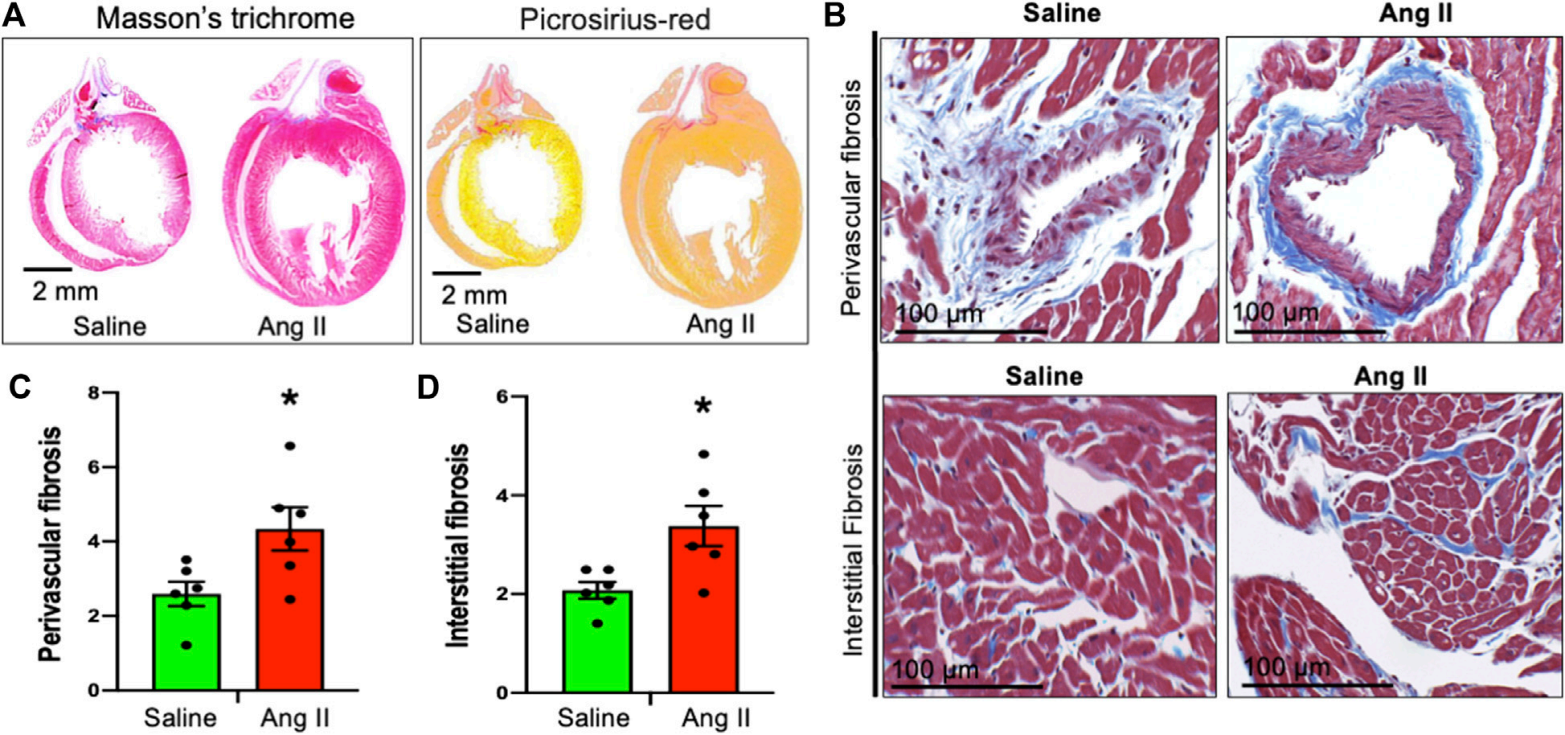
Ang II heart showed cardiac fibrosis. **(A)** Whole heart Masson’s trichrome (left) and Sirius red (right) staining of saline- and Ang II-treated rat hearts. **(B)** Representative Masson’s trichrome (blue) staining of heart longitudinal sections from saline- and Ang II-treated rats. **(C)** Quantifications of Masson’s trichrome perivascular blue intensity in saline- and Ang II-treated rat hearts. Each dot represents the mean collagen intensity from the left ventricular heart section (20 fields per fixed heart section; three sections/rat). **(D)** Quantifications of Masson’s trichrome interstitial blue intensity in saline- and Ang II-treated hearts. Each dot represents the mean collagen intensity from the left ventricular heart section (20 fields per fixed heart section; three sections/rat). Values are presented as mean ± SEM, n = 6. *p < 0.05 vs saline, Student’s t-test.

To monitor cardiac fibrosis, we performed Masson’s trichrome and Picrosirius staining of longitudinal cardiac sections, where Masson’s trichrome stained the collagen fiber as blue and Picrosirius stained the fiber as red in hearts of control and Ang II-infused rats (Figure 6A). Hearts from centrally Ang II-infused rats displayed an increase in both perivascular and interstitial fibrosis stained with either Masson’s trichrome or Picrosirius staining (Figures 6B–D). Together, these cardiac histological data indicate a progressive cardiac extracellular fibrosis and remodeling in rats infused with Ang II. Masson’s trichrome and Picrosirius red staining further confirmed that there was an enlargement of the left atrium; however, it should be noted that the overall atrial and right ventricle fibrosis staining was similar between the groups.

### Cardiac hypertrophy and inflammation

**FIGURE 7:**
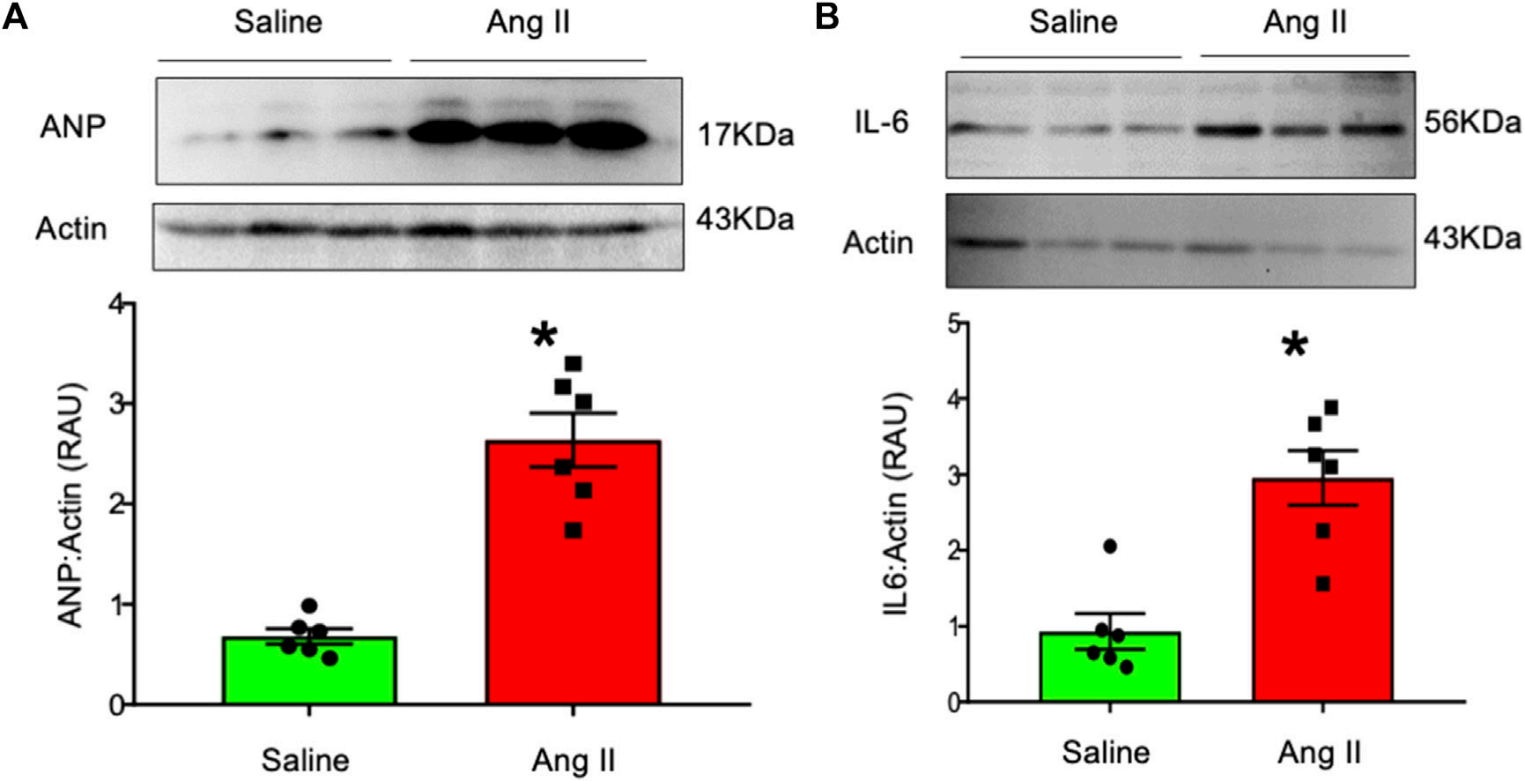
Western blot analysis of **(A)** atrial natriuretic factor (ANP) and **(B)** interleukin-6 (IL-6) in left ventricular tissues from saline- and Ang II-infused rats. The top panels show raw gel images of the proteins in ventricular tissues. The bottom panels show the composite data from each group. Values are presented as mean ± SEM, n = 6. *p < 0.05 vs saline, Student’s t-test.

To further characterize cardiac hypertrophy and inflammation, we determined ventricular levels of ANP and IL6 as markers for these conditions, respectively. Hearts from Ang II-infused rats demonstrated increased ANP and IL-6 levels in the LV tissues (Figure 7). These data indicate that there were increased markers for both cardiac hypertrophy and inflammation in rats infused with Ang II centrally.

### Morphological and functional aspects of HFpEF in Ang II-infused rats

Morphological aspects and functional aspects are used clinically to diagnose HFpEF (Reddy et al., 2018; Pfeffer et al., 2019; Pieske et al., 2019). The concept of a diagnostic algorithm that incorporates imaging and biomarkers (NPs) was advocated by the HFA (Williams et al., 2018) and adapted by other research workers (Reddy et al., 2018; Pfeffer et al., 2019; Pieske et al., 2019; Lam et al., 2020). Table 2 provides a list of morphological and functional aspects that are present in rats centrally infused with Ang II. Pieske et al. (2019) have used the “HFA-PEFF diagnostic algorithm” in the diagnostic process of HFpEF, where they have assigned major (2 points) and minor (1 point) criteria for each of the measures. In our study, we have conservatively assigned minor (1 point) for each of the features that were monitored and reported positive in this study, with a total HFA-PEFF score of 16. According to Pieske et al. (2019), a score of ≥5 points is considered to be diagnostic of HFpEF.

**TABLE 2:** Comparison of morphological and functional aspects of HFpEF model with HFA-PEFF score (Pieske et al., 2019).

Features of the model	Present	HFA-PEFF score points
*Morphological aspects*		
Left ventricular mass	+	1
Left atrial enlargement	+	1
Wall thickness	+	1
Concentric hypertrophy	+	1
Cardiac myocyte number	+	1
Cardiac myocyte diameter	+	1
Perivascular fibrosis	+	1
Interstitial fibrosis	+	1
*Functional aspects*		
Diastolic function	+	1
Reduced E/A	+	1
Reduced diastolic relaxation after isoproterenol administration	+	1
Increased LVEDP	+	1
Increased arterial blood pressure	+	1
Increased lung weight congestion	+	1
*Functional markers*		
Atrial natriuretic factor (ANP)	+	1
Interleukin-6 (IL-6)	+	1
≥5 point = HFpEF		16

## Discussion

Central infusion of Ang II resulted in neurogenic hypertension in rats that demonstrated elevated blood pressure, concentric LV hypertrophy, and increased LV fibrosis. These rats also demonstrated functionally preserved fractional shortening, with preserved ejection fraction but reduced diastolic function depicted by a reduced E/A ratio, increased LV filling pressure (LVEDP), and reduced relaxation of LV to an adrenergic challenge. These rats also exhibited left atrial enlargement, pulmonary congestion, and elevated levels of atrial natriuretic peptide and inflammatory marker IL6. Thus, neurogenic hypertension in these centrally Ang II-infused rats mimics the phenotype of HFpEF, independent of obesity, diabetes, or age. All these markers/measures which are characteristic hallmarks of clinical HFpEF (Table 2) (Reddy et al., 2018; Pfeffer et al., 2019; Pieske et al., 2019) are recapitulated in this model of neurogenic hypertension in rats (Nandi et al., 2021; Sharma et al., 2021).

Previously, we have shown that central infusion of Ang II for 14 days resulted in an increase in sympatho-excitation, leading to hypertension in this model of neurogenic hypertension in rats (Nandi et al., 2021; Sharma et al., 2021). In previous studies, we have demonstrated that Ang II-infused rats had a specifically higher cardiac sympathetic tone accompanied by a reduced parasympathetic drive than saline-infused rats. The balance between cardiac sympathetic and parasympathetic tones, evaluated by the ratio between these variables, was almost 2.5 times higher in Ang II-infused rats, showing that Ang II infusion leads to an overwhelming cardiac autonomic imbalance favoring sympatho-excitation and consequent tachycardia. It has been suggested that centrally induced sympatho-excitation that elicits a cardiac sympatho-excitation with concomitant hypertension leads to associated cardiac and metabolic alterations (Kline et al., 1983; Schohn et al., 1985; Patel et al., 2016; Borovac et al., 2020). This model of centrally mediated sympatho-excitation by central infusion of Ang II leading to hypertension recapitulates most features of clinical HFpEF as hypertension has been observed in more than three quartiles of human HFpEF patients (He et al., 2021; Pagel et al., 2021). It should be noted that we used a very low dose of Ang II infusion given ICV to elicit activation of central sympatho-excitatory mechanisms within the brain that is sufficient to result in neurogenic systemic hypertension (Sharma et al., 2021). Furthermore, this dose of Ang II when given systemically (in the general circulation, IV) would be insufficient to elicit a pressor response. The present model, therefore, differs from prior models that used intravenous ANG II infusion by the absence of direct effects of ANG II on the vasculature (vasocontraction and subsequent hypertension), as well as the heart itself (hypertrophy), or metabolic abnormalities, and as such, it is likely to provide information that is not available from the other models, yet it is complementary to the already available models. We posit that we avoided mimicking systemic hypertension that would be elicited by direct actions of peripheral administration of Ang II or direct effects of Ang II on the heart (Regan et al., 2015), as targeting this form of systemic hypertension has failed in clinical trials (Pitt et al., 2014). Our current model takes advantage of the fact that such a specific central manipulation using central Ang II mechanisms results in cardiac sympatho-excitation with concomitant hypertension that is often observed in HFpEF with local cardiac remodeling (Sone et al., 1984; Hoenig et al., 2008). We are presuming that we have a neurogenic form of hypertension caused by an enhanced sympathetic drive (which may or may not include volume expansion).

Clinically, heart failure with preserved ejection fraction (HFpEF) is a complex syndrome with multifactorial disease heterogeneity, and there is a lack of a suitable preclinical murine model for HFpEF, as it is practically impossible to introduce all associated comorbidities in a single model to mimic an advanced clinical HFpEF. In this study, we attempted to describe the phenotype of HFpEF in a model of centrally induced neurogenic sympatho-excitation, independent of obesity, diabetes, or age. This model of neurogenic hypertension would assist in elucidating the possible contributions of other factors, such as renal dysfunction due to hyperfiltration or baroreceptor dysfunction independent of sympatho-excitation.

Our current model takes advantage of the fact that such a specific central manipulation results in cardiac sympatho-excitation with concomitant hypertension that is often mimicked in HFpEF with local cardiac remodeling (Sone et al., 1984; Hoenig et al., 2008). Using noninvasive pulse-wave Doppler and M-mode echocardiography, we observed that Ang II-infused rats demonstrated a decrease in E/A with preserved ejection fraction. Furthermore, using invasive hemodynamic measurements, we observed an elevated LVEDP which is considered the gold standard to monitor diastolic dysfunction indicative of HFpEF. Although E/e’ was not significantly increased in this study, it should be noted that the E/e’ ratio is typically only observed to be elevated when the LVEDP is substantially increased (>20 mmHg) in patients with HFpEF (Oh et al., 2011). Because the increase in LVEDP in our study was in the range of 5 mmHg, it was perhaps not high enough to elicit a significant increase in E/e’. In addition, although there was a normal contractile response to isoproterenol, the relaxation response to isoproterenol was significantly blunted in rats infused with Ang II, suggesting diastolic dysfunction. Taken together, these results suggest that the Ang II-infused rats have impaired diastolic function similar to commonly observed diastolic dysfunction in clinical HFpEF.

Cardiac fibrosis is often observed in patients with HFpEF as a primary event to supplement cardiac hypertrophic expansion (Hahn et al., 2021). Further examination of the fibrosis in the hearts of patients with HFpEF has revealed that collagen fibers are deposited in cardiac perivascular and interstitial spaces, often termed as perivascular or interstitial fibrosis. Using anatomical and histological approaches, we observed similar cardiac LV hypertrophy, left atrial enlargement, and LV cardiac fibrosis, both in the perivascular and interstitial spaces, which is indicative of a myriad of cardiac remodeling in rats infused with Ang II. It is of importance to note that increased left atrial size is a common clinical feature of HFpEF patients and partially contributes to increased atrial fibrillation incidence.

The presence of enhanced sympatho-excitation in this model appears to be a fundamental component for the genesis of cardiac pathophysiology, which is similar to that observed in patients with HFpEF. Therefore, in this unique model, we observed LV diastolic dysfunction, as evidenced by a significant decrease in diastolic early to late transmittal filling velocities, an elevated LVEDP, and a lack of appropriate relaxation during an isoproterenol challenge. This was linked with intense myocardial hypertrophy and fibrosis, yet a preserved cardiac ejection fraction. Our results provide a unique murine model that exhibits many of the features of HFpEF in rats which can be utilized for testing therapeutic strategies in future translational studies. Therefore, strategies for reducing the activation of sympathetic outflow either centrally or peripherally in the appropriate amount, specifically, may provide novel approaches for the development of future therapeutic agents for HFpEF (Schohn et al., 1985; Nikami et al., 2005). Of note, HFpEF is a complex syndrome with multifactorial disease heterogeneity, and currently, there is a lack of a suitable preclinical rat model of HFpEF, as it is practically impossible to introduce all associated comorbidities in a single model to mimic advanced clinical HFpEF.

It should be noted that although several other complex cardiac and non-cardiac or vascular issues are commonly observed in clinical setting, including atrial fibrillation, arterial stiffness, enlarged left atrium, increased LV mass, altered atrial–ventricular coupling, skeletal muscle dysfunction, decreased intra-myocardial capillary density, impaired active vasoconstriction, and cardiac relaxation problems that are frequently observed in patients with HFpEF (Volpe et al., 2016; Messerli et al., 2017; Davila et al., 2019; Bode et al., 2020; Schauer et al., 2020), our unique model mirrors a majority of these abnormalities. It should also be noted that some of these abnormalities are in the slight to moderate level, indicative of either a beginning stage or a milder form of the disease.

We acknowledge that the pathophysiology of diastolic dysfunction in HFpEF is complex to understand entirely at this point; however, we believe that identifying a viable murine model for this condition will enable future studies to elucidate the underlying mechanisms for HFpEF as well as explore effective treatment for HFpEF patients. Albeit this is a very compelling model of HFpEF, we acknowledge some potential limitations of our study: first, it is challenging to model a preclinical animal model that mimics all basic clinical features of HFpEF in a single pathophysiology model. However, we explored a possible contribution of a central mechanism (enhanced cardiac sympatho-excitation) that may be responsible for HFpEF cardiac phenotypic changes at the cell, tissue, and anatomical levels. Second, exercise intolerance: a reduction in the ability to endure maximum exercise workload during the exercise period is one of the hallmark features of clinical HFpEF that were not tested in this study. We believe that although exercise tolerance parameters are useful functional endpoints, the lack of exercise intolerance data does not exclude or diminish the importance of all the key aspects of HFpEF examined in this study. We did observe indices of pulmonary congestion, which were thought to be in part responsible for exercise intolerance. We also observed reduced relaxation response to an acute challenge with isoproterenol. Third, although patients with HFpEF often present clinically with comorbidities like central obesity and metabolic disorders, these disorders are also invariably accompanied by sympatho-excitation, as presented in the current study. Fourth, in patients with HFpEF, the LVEDP is typically significantly higher than our observations in this study. However, it should be noted that in our model, LVEDP was typically measured under anesthetized conditions, acutely with a 14-day infusion of ICV Ang II. This may perhaps be due to the shorter duration of treatment. Therefore, a longer period of sympatho-excitation with a longer period of Ang II infusion would be expected to increase LVEDP similar to that observed in clinical HFpEF.

## Perspectives

This study demonstrates that specific central activation of the sympathetic nervous system recapitulates a majority of the features of HFpEF, namely, elevated LVEDP, impaired LV relaxation, pathological cardiac concentric hypertrophy, cardiac fibrosis, and elevated levels of ANP and IL-6 in the absence of LV systolic dysfunction, and may, therefore, be considered as a novel tool for use in mechanistic preclinical studies in HFpEF. This novel rat model of neurogenic sympathetic overactivation appears to be fundamentally involved in the genesis and the progression of HFpEF. This model may be important to study HFpEF as a preclinical research tool with the potential inclusion of multi-organ failure and metabolic insult incorporation as additional factors that can be examined to identify key molecular mechanisms to elucidate potential therapeutic strategies for the treatment of HFpEF.

## Data Availability

The original contributions presented in the study are included in the article/Supplementary Material; further inquiries can be directed to the corresponding author.

## References

[B1] Aikawa T. Naya M. Obara M. Manabe O. Tomiyama Y. Magota K. (2017). Impaired myocardial sympathetic innervation is associated with diastolic dysfunction in heart failure with preserved ejection fraction: (11)C-hydroxyephedrine PET study. J. Nucl. Med. 58 (5), 784–790. 10.2967/jnumed.116.178558 27811122

[B2] Altara R. Giordano M. Norden E. S. Cataliotti A. Kurdi M. Bajestani S. N. (2017). Targeting obesity and diabetes to treat heart failure with preserved ejection fraction. Front. Endocrinol. (Lausanne) 8, 160. 10.3389/fendo.2017.00160 28769873 PMC5512012

[B3] Barki-Harrington L. Perrino C. Rockman H. A. (2004). Network integration of the adrenergic system in cardiac hypertrophy. Cardiovasc Res. 63 (3), 391–402. 10.1016/j.cardiores.2004.03.011 15276464

[B4] Becker B. K. Wang H. Zucker I. H. (2017). Central TrkB blockade attenuates ICV angiotensin II-hypertension and sympathetic nerve activity in male Sprague-Dawley rats. Auton. Neurosci. 205, 77–86. 10.1016/j.autneu.2017.05.009 28549782 PMC5516943

[B5] Bode D. Wen Y. Hegemann N. Primessnig U. Parwani A. Boldt L. H. (2020). Oxidative stress and inflammatory modulation of Ca(2+) handling in metabolic HFpEF-related left atrial cardiomyopathy. Antioxidants (Basel) 9 (9), 860. 10.3390/antiox9090860 32937823 PMC7555173

[B6] Borovac J. A. D'Amario D. Bozic J. Glavas D. (2020). Sympathetic nervous system activation and heart failure: current state of evidence and the pathophysiology in the light of novel biomarkers. World J. Cardiol. 12 (8), 373–408. 10.4330/wjc.v12.i8.373 32879702 PMC7439452

[B7] Camara A. K. Osborn J. L. (1999). Alpha-adrenergic systems mediate chronic central AII hypertension in rats fed high sodium chloride diet from weaning. J. Auton. Nerv. Syst. 76 (1), 28–34. 10.1016/s0165-1838(99)00003-x 10323304

[B8] Davila A. Tian Y. Czikora I. Li J. Su H. Huo Y. (2019). Adenosine kinase inhibition augments conducted vasodilation and prevents left ventricle diastolic dysfunction in heart failure with preserved ejection fraction. Circ. Heart Fail 12 (8), e005762. 10.1161/CIRCHEARTFAILURE.118.005762 31525084 PMC6750030

[B9] Deng Y. Xie M. Li Q. Xu X. Ou W. Zhang Y. (2021). Targeting mitochondria-inflammation circuit by beta-hydroxybutyrate mitigates HFpEF. Circ. Res. 128 (2), 232–245. 10.1161/CIRCRESAHA.120.317933 33176578

[B10] Dikalov S. I. Nazarewicz R. R. (2013). Angiotensin II-induced production of mitochondrial reactive oxygen species: potential mechanisms and relevance for cardiovascular disease. Antioxid. Redox Signal 19 (10), 1085–1094. 10.1089/ars.2012.4604 22443458 PMC3771548

[B11] Fonkoue I. T. Le N. A. Kankam M. L. DaCosta D. Jones T. N. Marvar P. J. (2019). Sympathoexcitation and impaired arterial baroreflex sensitivity are linked to vascular inflammation in individuals with elevated resting blood pressure. Physiol. Rep. 7 (7), e14057. 10.14814/phy2.14057 30968587 PMC6456445

[B12] Gori M. Iacovoni A. Senni M. (2016). Haemodynamics of heart failure with preserved ejection fraction: a clinical perspective. Card. Fail Rev. 2 (2), 102–105. 10.15420/cfr.2016:17:2 28785461 PMC5490949

[B13] Hahn V. S. Knutsdottir H. Luo X. Bedi K. Margulies K. B. Haldar S. M. (2021). Myocardial gene expression signatures in human heart failure with preserved ejection fraction. Circulation 143 (2), 120–134. 10.1161/CIRCULATIONAHA.120.050498 33118835 PMC7856095

[B14] Hanff T. C. Cohen J. B. Zhao L. Javaheri A. Zamani P. Prenner S. B. (2021). Quantitative proteomic analysis of diabetes mellitus in heart failure with preserved ejection fraction. JACC Basic Transl. Sci. 6 (2), 89–99. 10.1016/j.jacbts.2020.11.011 33665511 PMC7907637

[B15] He J. Sirajuddin A. Li S. Zhuang B. Xu J. Zhou D. (2021). Heart failure with preserved ejection fraction in hypertension patients: a myocardial mr strain study. J. Magn. Reson Imaging 53 (2), 527–539. 10.1002/jmri.27313 32896042

[B16] Hoenig M. R. Bianchi C. Rosenzweig A. Sellke F. W. (2008). The cardiac microvasculature in hypertension, cardiac hypertrophy and diastolic heart failure. Curr. Vasc. Pharmacol. 6 (4), 292–300. 10.2174/157016108785909779 18855717

[B17] Kalil G. Z. Haynes W. G. (2012). Sympathetic nervous system in obesity-related hypertension: mechanisms and clinical implications. Hypertens. Res. 35 (1), 4–16. 10.1038/hr.2011.173 22048570 PMC3902842

[B18] Kaye D. Esler M. (2005). Sympathetic neuronal regulation of the heart in aging and heart failure. Cardiovasc Res. 66 (2), 256–264. 10.1016/j.cardiores.2005.02.012 15820194

[B19] Kaye D. M. Lambert G. W. Lefkovits J. Morris M. Jennings G. Esler M. D. (1994). Neurochemical evidence of cardiac sympathetic activation and increased central nervous system norepinephrine turnover in severe congestive heart failure. J. Am. Coll. Cardiol. 23 (3), 570–578. 10.1016/0735-1097(94)90738-2 8113536

[B20] Kline R. L. Patel K. P. Ciriello J. Mercer P. F. (1983). Effect of renal denervation on arterial pressure in rats with aortic nerve transection. Hypertension 5, 468–475. 10.1161/01.hyp.5.4.468 6134668

[B21] Lam C. S. P. Voors A. A. Piotr P. McMurray J. J. V. Solomon S. D. (2020). Time to rename the middle child of heart failure: heart failure with mildly reduced ejection fraction. Eur. Heart J. 41 (25), 2353–2355. 10.1093/eurheartj/ehaa158 32227233

[B22] Little W. C. (2008). Hypertension, heart failure, and ejection fraction. Circulation 118 (22), 2223–2224. 10.1161/CIRCULATIONAHA.108.819318 19001020

[B23] Loredo-Mendoza M. L. Ramirez-Sanchez I. Bustamante-Pozo M. M. Ayala M. Navarrete V. Garate-Carrillo A. (2020). The role of inflammation in driving left ventricular remodeling in a pre-HFpEF model. Exp. Biol. Med. (Maywood) 245 (8), 748–757. 10.1177/1535370220912699 32183553 PMC7221483

[B24] Messerli F. H. Rimoldi S. F. Bangalore S. (2017). The transition from hypertension to heart failure: contemporary update. JACC Heart Fail 5 (8), 543–551. 10.1016/j.jchf.2017.04.012 28711447

[B25] Nandi S. S. Katsurada K. Mahata S. K. Patel K. P. (2021). Neurogenic hypertension mediated mitochondrial abnormality leads to cardiomyopathy: contribution of UPR^mt^ and norepinephrine-miR- 18a-5p-HIF-1α Axis. Front. Physiol. 12, 718982. 10.3389/fphys.2021.718982 34912235 PMC8667690

[B26] Nandi S. S. Katsurada K. Sharma N. M. Anderson D. R. Mahata S. K. Patel K. P. (2020). MMP9 inhibition increases autophagic flux in chronic heart failure. Am. J. Physiol. Heart Circ. Physiol. 319 (6), H1414–H1437. 10.1152/ajpheart.00032.2020 33064567 PMC7792705

[B27] Nandi S. S. Zheng H. Sharma N. M. Shahshahan H. R. Patel K. P. Mishra P. K. (2016). Lack of miR-133a decreases contractility of diabetic hearts: a role for novel cross talk between tyrosine aminotransferase and tyrosine hydroxylase. Diabetes 65, 3075–3090. 10.2337/db16-0023 27411382 PMC5033264

[B28] Nguyen I. T. N. Brandt M. M. van de Wouw J. van Drie R. W. A. Wesseling M. Cramer M. J. (2020). Both male and female obese ZSF1 rats develop cardiac dysfunction in obesity-induced heart failure with preserved ejection fraction. PLoS One 15 (5), e0232399. 10.1371/journal.pone.0232399 32374790 PMC7202634

[B29] Nikami H. Nedergaard J. Fredriksson J. M. (2005). Norepinephrine but not hypoxia stimulates HIF-1alpha gene expression in brown adipocytes. Biochem. Biophys. Res. Commun. 337 (1), 121–126. 10.1016/j.bbrc.2005.09.011 16171784

[B30] Oh J. K. Park S. J. Nagueh S. F. (2011). Established and novel clinical applications of diastolic function assessment by echocardiography. Circ. Cardiovasc Imaging 4 (4), 444–455. 10.1161/CIRCIMAGING.110.961623 21772012

[B31] Olver T. D. Edwards J. C. Jurrissen T. J. Veteto A. B. Jones J. L. Gao C. (2019). Western diet-fed, aortic-banded ossabaw swine: a preclinical model of cardio-metabolic heart failure. JACC Basic Transl. Sci. 4 (3), 404–421. 10.1016/j.jacbts.2019.02.004 31312763 PMC6610000

[B32] Pagel P. S. Tawil J. N. Boettcher B. T. Izquierdo D. A. Lazicki T. J. Crystal G. J. (2021). Heart failure with preserved ejection fraction: a comprehensive review and update of diagnosis, pathophysiology, treatment, and perioperative implications. J. Cardiothorac. Vasc. Anesth. 35 (6), 1839–1859. 10.1053/j.jvca.2020.07.016 32747202

[B33] Patel K. P. Xu B. Liu X. Sharma N. M. Zheng H. (2016). Renal denervation improves exaggerated sympathoexcitation in rats with heart failure: a role for neuronal nitric oxide synthase in the paraventricular nucleus. Hypertension 68 (1), 175–184. 10.1161/HYPERTENSIONAHA.115.06794 27185748 PMC4900899

[B34] Paulus W. J. Tschope C. (2013). A novel paradigm for heart failure with preserved ejection fraction: comorbidities drive myocardial dysfunction and remodeling through coronary microvascular endothelial inflammation. J. Am. Coll. Cardiol. 62 (4), 263–271. 10.1016/j.jacc.2013.02.092 23684677

[B35] Paxinos G. Watson C. R. Emson P. C. (1980). AChE-stained horizontal sections of the rat brain in stereotaxic coordinates. J. Neurosci. Methods 3 (2), 129–149. 10.1016/0165-0270(80)90021-7 6110810

[B36] Pfeffer M. A. Shah A. M. Borlaug B. A. (2019). Heart failure with preserved ejection fraction in perspective. Circ. Res. 124 (11), 1598–1617. 10.1161/CIRCRESAHA.119.313572 31120821 PMC6534165

[B37] Pieske B. Tschope C. de Boer R. A. Fraser A. G. Anker S. D. Donal E. (2019). How to diagnose heart failure with preserved ejection fraction: the HFA-PEFF diagnostic algorithm: a consensus recommendation from the Heart Failure Association (HFA) of the European Society of Cardiology (ESC). Eur. Heart J. 40 (40), 3297–3317. 10.1093/eurheartj/ehz641 31504452

[B38] Pitt B. Pfeffer M. A. Assmann S. F. Boineau R. Anand I. S. Claggett B. (2014). Spironolactone for heart failure with preserved ejection fraction. N. Engl. J. Med. 370 (15), 1383–1392. 10.1056/NEJMoa1313731 24716680

[B39] Reddy Y. N. V. Carter R. E. Obokata M. Redfield M. M. Borlaug B. A. (2018). A simple, evidence-based approach to help guide diagnosis of heart failure with preserved ejection fraction. Circulation 138 (9), 861–870. 10.1161/CIRCULATIONAHA.118.034646 29792299 PMC6202181

[B40] Regan J. A. Mauro A. G. Carbone S. Marchetti C. Gill R. Mezzaroma E. (2015). A mouse model of heart failure with preserved ejection fraction due to chronic infusion of a low subpressor dose of angiotensin II. Am. J. Physiol. Heart Circ. Physiol. 309 (5), H771–H778. 10.1152/ajpheart.00282.2015 26188021 PMC4591411

[B41] Sava R. I. Pepine C. J. March K. L. (2020). Immune dysregulation in HFpEF: a target for mesenchymal stem/stromal cell therapy. J. Clin. Med. 9 (1), 241. 10.3390/jcm9010241 31963368 PMC7019215

[B42] Schauer A. Draskowski R. Jannasch A. Kirchhoff V. Goto K. Mannel A. (2020). ZSF1 rat as animal model for HFpEF: development of reduced diastolic function and skeletal muscle dysfunction. Esc. Heart Fail 7 (5), 2123–2134. 10.1002/ehf2.12915 32710530 PMC7524062

[B43] Schiattarella G. G. Altamirano F. Tong D. French K. M. Villalobos E. Kim S. Y. (2019). Nitrosative stress drives heart failure with preserved ejection fraction. Nature 568 (7752), 351–356. 10.1038/s41586-019-1100-z 30971818 PMC6635957

[B44] Schohn D. Weidmann P. Jahn H. Beretta-Piccoli C. (1985). Norepinephrine-related mechanism in hypertension accompanying renal failure. Kidney Int. 28 (5), 814–822. 10.1038/ki.1985.203 4087696

[B45] Sharma N. M. Haibara A. S. Katsurada K. Nandi S. S. Liu X. Zheng H. (2021). Central Ang II (angiotensin II)-Mediated sympathoexcitation: role for HIF-1α (Hypoxia-Inducible factor-1α) facilitated glutamatergic tone in the paraventricular nucleus of the hypothalamus. Hypertension 77 (1), 147–157. 10.1161/HYPERTENSIONAHA.120.16002 33296248 PMC7720881

[B46] Sharp T. E. 3rd Scarborough A. L. Li Z. Polhemus D. J. Hidalgo H. A. Schumacher J. D. (2021). Novel gottingen miniswine model of heart failure with preserved ejection fraction integrating multiple comorbidities. JACC Basic Transl. Sci. 6 (2), 154–170. 10.1016/j.jacbts.2020.11.012 33665515 PMC7907541

[B47] Sone T. Miyazaki Y. Ogawa K. Satake T. (1984). Effects of excessive noradrenaline on cardiac mitochondrial calcium transport and oxidative phosphorylation. Jpn. Circ. J. 48 (5), 492–497. 10.1253/jcj.48.492 6727029

[B48] Upadhya B. Haykowsky M. J. Eggebeen J. Kitzman D. W. (2015). Exercise intolerance in heart failure with preserved ejection fraction: more than a heart problem. J. Geriatr. Cardiol. 12 (3), 294–304. 10.11909/j.issn.1671-5411.2015.03.013 26089855 PMC4460174

[B49] Volpe M. Santolamazza C. Tocci G. (2016). Hypertension in patients with heart failure with reduced ejection fraction. Curr. Cardiol. Rep. 18 (12), 127. 10.1007/s11886-016-0807-9 27796865

[B50] Williams B. Mancia G. Spiering W. Agabiti Rosei E. Azizi M. Burnier M. (2018). 2018 ESC/ESH Guidelines for the management of arterial hypertension. Eur. Heart J. 39 (33), 3021–3104. 10.1093/eurheartj/ehy339 30165516

[B51] Yoon S. Kim M. Lee H. Kang G. Bedi K. Margulies K. B. (2021). S-nitrosylation of histone deacetylase 2 by neuronal nitric oxide synthase as a mechanism of diastolic dysfunction. Circulation 143 (19), 1912–1925. 10.1161/CIRCULATIONAHA.119.043578 33715387

[B52] Zheng H. Liu X. Sharma N. M. Patel K. P. (2016). Renal denervation improves cardiac function in rats with chronic heart failure: effects on expression of β-adrenoceptors. Am. J. Physiol. Heart Circ. Physiol. 311 (2), H337–H346. 10.1152/ajpheart.00999.2015 27288440 PMC5504438

