# Supplementary material for: Enhanced central sympathetic tone induces heart failure with preserved ejection fraction (HFpEF) in rats
Source: Front Physiol. 2023 Dec 7;14:1277065. doi: 10.3389/fphys.2023.1277065 (PMC10758618; doi:10.3389/fphys.2023.1277065)
Supplement: Supplementary file 1 [file Presentation1.PPTX]

## Slide 1
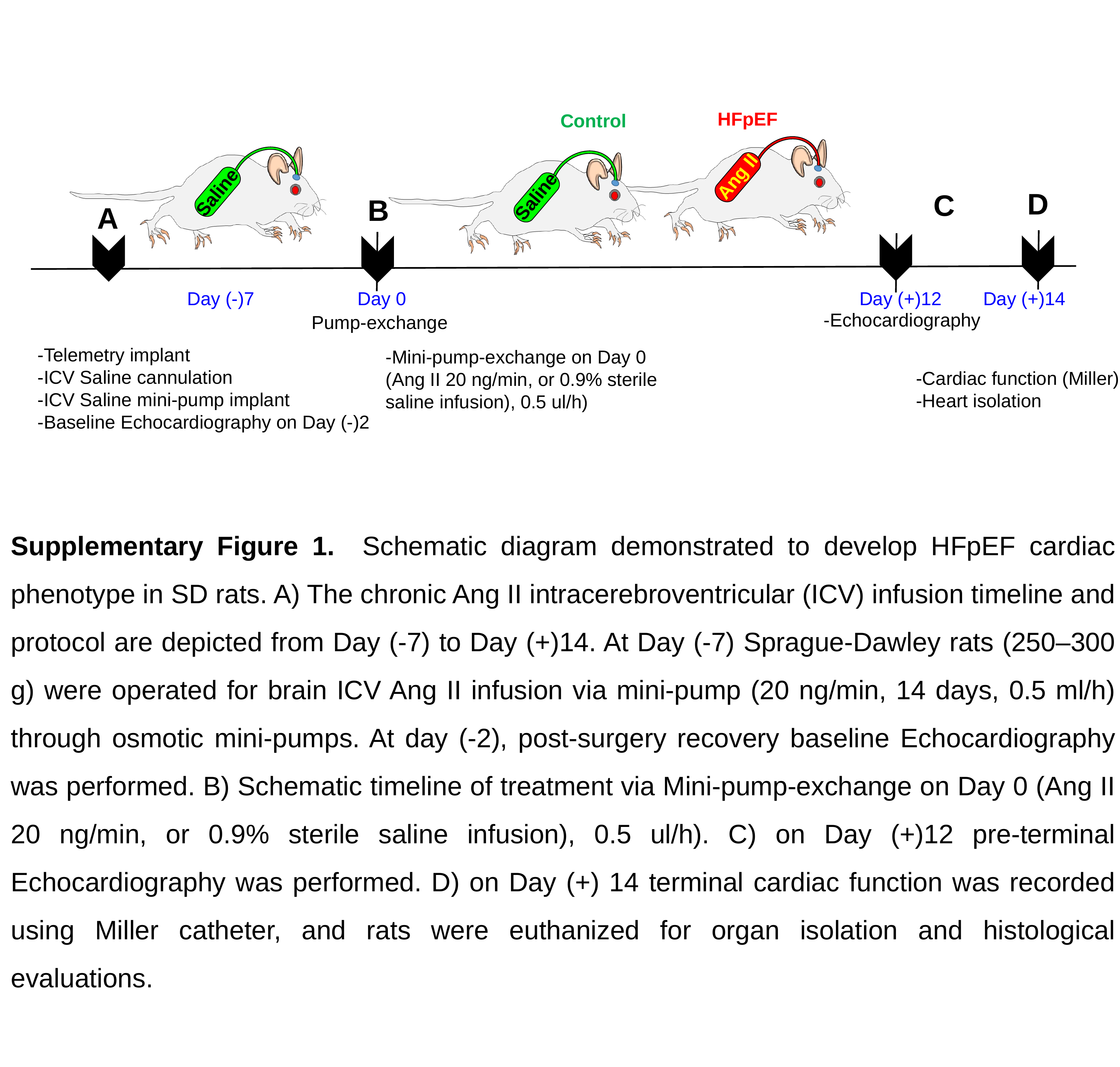

HFpEF
Control
Ang II
Saline
Saline
D
C
B
A
Day (-)7 Day 0 Day (+)12 Day (+)14
-Echocardiography
Pump-exchange
-Telemetry implant
-ICV Saline cannulation
-ICV Saline mini-pump implant
-Baseline Echocardiography on Day (-)2
-Mini-pump-exchange on Day 0
(Ang II 20 ng/min, or 0.9% sterile
saline infusion), 0.5 ul/h)
-Cardiac function (Miller)
-Heart isolation
Supplementary Figure 1. Schematic diagram demonstrated to develop HFpEF cardiac phenotype in SD rats. A) The chronic Ang II intracerebroventricular (ICV) infusion timeline and protocol are depicted from Day (-7) to Day (+)14. At Day (-7) Sprague-Dawley rats (250–300 g) were operated for brain ICV Ang II infusion via mini-pump (20 ng/min, 14 days, 0.5 ml/h) through osmotic mini-pumps. At day (-2), post-surgery recovery baseline Echocardiography was performed. B) Schematic timeline of treatment via Mini-pump-exchange on Day 0 (Ang II 20 ng/min, or 0.9% sterile saline infusion), 0.5 ul/h). C) on Day (+)12 pre-terminal Echocardiography was performed. D) on Day (+) 14 terminal cardiac function was recorded using Miller catheter, and rats were euthanized for organ isolation and histological evaluations.
